# Physical activity and advanced fibrosis in MASLD, MetALD, and ALD in a nationally representative cohort: NHANES 2017–2020

**DOI:** 10.1097/HC9.0000000000000797

**Published:** 2025-10-14

**Authors:** Nicholas Dunn, Wanyu Zhang, Hanna L. Blaney, Luis Antonio Diaz, Arpan Patel, Robert J. Wong, Juan Pablo Arab, Zobair Younossi, Craig McClain, Maiying Kong, Ashwani K. Singal

**Affiliations:** 1 Pembroke Hill School, Kansas City, Missouri, USA; 2 Department of Bioinformatics and Biostatistics, University of Louisville, Louisville, Kentucky, USA; 3 Division of GI and Hepatology, Georgetown University, Washington, District of Columbia, USA; 4 Division of GI and Hepatology, University of California San Diego, San Diego, California, USA; 5 Division of GI and Hepatology, University of California Los Angeles, Los Angeles, California, USA; 6 Division of Gastroenterology and Hepatology, Stanford University School of Medicine, Palo Alto, California, USA; 7 Department of Internal Medicine, Division of Gastroenterology, Hepatology, and Nutrition, Stravitz-Sanyal Institute for Liver Disease and Metabolic Health, Virginia Commonwealth University School of Medicine, Richmond, Virginia, USA; 8 The Global NASH/MASH Council, Washington, District of Columbia, USA; 9 Department of Medicine, University of Louisville School of Medicine, Louisville, Kentucky, USA; 10 Department of Medicine, Rob Rexley VA Medical Center, Louisville, Kentucky, USA; 11 University of Louisville Health Jewish Hospital and Trager Transplant Center, Louisville, Kentucky, USA

**Keywords:** ALD, cirrhosis, MASLD, MetALD, steatosis

## Abstract

**Background:**

It is unclear whether physical activity (PA) and advanced fibrosis are associated in alcohol-associated liver disease (ALD) and metabolic dysfunction and alcohol-associated liver disease (MetALD). We examined the association between work-related PA (WPA) or leisure-time PA (LTPA) and advanced fibrosis across the steatotic liver disease (SLD) spectrum.

**Methods:**

Data obtained from the National Health and Nutrition Examination Survey (2017-2020) in adults with hepatic steatosis were included and categorized into MASLD (n=2236), MetALD (n=1355), and ALD (n=457) based on alcohol use. PA was quantified as metabolic equivalent (MET)-minutes per week, and ≥600 MET-min/wk was used as the threshold for high activity. Adjusted logistic regression models for confounders (sex, race/ethnicity, education, income, metabolic risk factors, and etiology of SLD) were used to analyze the relationship of PA types with at-risk advanced fibrosis, according to an Agile 3+ score ≥0.45.

**Results:**

Among 4342 SLD participants, LTPA was strongly linked to a lower risk of at-risk advanced fibrosis in MASLD (OR=0.56, 95% CI: 0.34–0.93), as well as MetALD and ALD combined (OR=0.55, 95% CI: 0.34–0.89). WPA was not associated with improved advanced fibrosis. There were no interactions between WPA and SLD subtypes (*p*=0.98), and between LTPA and SLD subtypes (*p*=0.55).

**Conclusions:**

LTPA but not WPA is protective for at-risk advanced fibrosis in patients with MASLD and MetALD. These findings showcase the importance of structured LTPA interventions to mitigate fibrosis risk in SLD. Larger studies are needed to examine the benefits of PA in patients with ALD and to delineate exercise prescriptions for patients with SLD.

## INTRODUCTION

Physical activity (PA) has been widely recognized as a key component in the treatment of metabolic disorders, such as metabolic dysfunction–associated steatotic liver disease (MASLD), formerly known as NAFLD. Empirical evidence indicates that recreational or leisure-time PA (LTPA), particularly aerobic and resistance exercise, is associated with reduced hepatic steatosis, improved insulin sensitivity, and decreased risks of advanced fibrosis and death in individuals with MASLD.^1^^–^^5^ However, the effect of PA on the broader range of steatotic liver disease (SLD), such as metabolic dysfunction and alcohol-associated liver disease (MetALD) and alcohol-associated liver disease (ALD), is unknown. Furthermore, the contribution of work-related PA (WPA) to liver health has not been examined in a systematic way despite its interest in an occupational setting.

PA regulates the major biologic metabolic pathways, such as hepatic lipid metabolism, inflammation, and mitochondrial function, thereby resulting in improvements in hepatic triglyceride content and liver histology independent of weight loss.^1^ In addition, PA exerts systemic influences through enhancing cardiorespiratory fitness, insulin resistance, and lipid homeostasis, all of which play an important role in the pathogenesis of SLD. Prospective cohort studies have demonstrated that LTPA is linked to reduced all-cause mortality in MASLD, with vigorous activity conferring additional survival benefit.^2^^,^^5^^,^^6^ Whether these results apply to MetALD and ALD, where alcohol-associated hepatotoxicity may neutralize some of the beneficial effects of PA, has yet to be determined.

Aside from LTPA, the role of WPA in liver disease has not been reviewed. Whereas LTPA, which is structured, is usually followed by beneficial health effects, the effect of WPA is less certain. WPA can entail prolonged standing, frequent movement, and exertion. It is unclear if this type of PA has similar metabolic beneficial effects. Limited research indicates that WPA is neutral or even harmful to health because of heightened energy expenditure without adequate recovery, stress, or compensation mechanisms like elevated caloric intake. As such, it is important to consider whether WPA exerts similar preventive benefits on liver fibrosis and SLD spectrum disease progression.

Our aim was to evaluate the association of both WPA and LTPA with prevalent advanced fibrosis in MASLD, MetALD, and ALD through the National Health and Nutrition Examination Survey (NHANES) 2017–2020 data. Specifically, we (1) examined if LTPA is related to lower fibrosis risk by SLD subtype; (2) examined WPA effects on the severity of liver disease; and (3) examined PA type, intensity, and SLD subtype interaction. By addressing these knowledge gaps, our findings will contribute to a fuller understanding of PA as a modifiable risk factor for liver fibrosis, informing targeted public health initiatives and clinical guidelines for patients across the whole SLD spectrum.

## METHODS

### Study population

**FIGURE 1:**
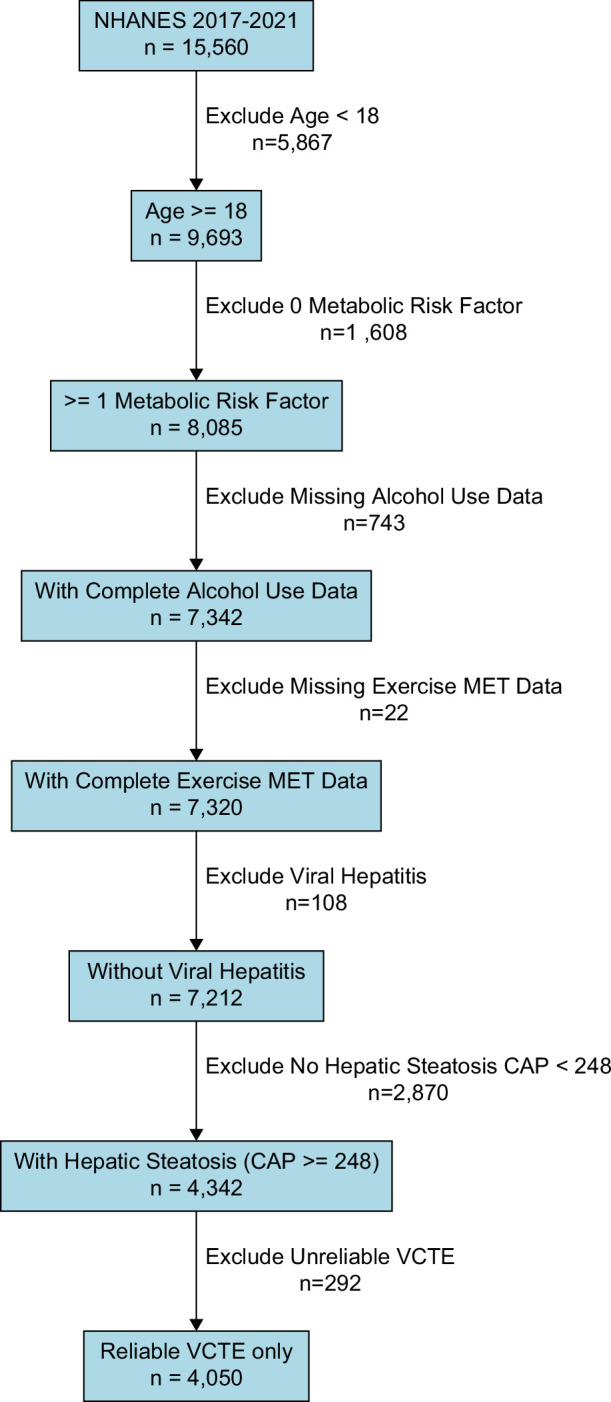
Study population attrition diagram from NHANES 2017–2020.

This study used data from the 2017–March 2020 pre-pandemic NHANES database. We included adults (≥18 y) with reliable elastography assessments. Exclusion criteria included fasting duration of <3 hours, fewer than 10 valid liver stiffness measurements (LSMs), and an IQR/M ratio >30%. We included participants with liver steatosis (controlled attenuation parameter >248 dB/m).^7^ and at least 1 cardiometabolic risk factor (CMRF). Subjects with missing information on alcohol use or PA and those positive for active HBV or HCV infection were excluded (Figure 1). The SLD population was classified into 3 groups based on alcohol consumption.^8^ Participants who answered “No” to ALQ111 (“Ever had a drink of any kind of alcohol”) or “Never in the last year” to ALQ121 (“Past 12 mo, how often drink alcoholic beverages”) were classified as MASLD. For participants reporting alcohol consumption, average daily intake (in grams) was calculated by dividing the number of drinks reported per day (ALQ130) by the frequency of drinking (ALQ121), assuming 14 g of alcohol per drink. Men consuming 30–60 g/d and women consuming 20–50 g/d were classified as MetALD, while those below these thresholds were classified as MASLD and those exceeding these thresholds were classified as ALD. The link to the detailed questionnaire is https://wwwn.cdc.gov/Nchs/Data/Nhanes/Public/2017/DataFiles/ALQ_J.htm

### Outcomes

The primary outcome was at-risk advanced fibrosis, defined as an Agile 3+ score ≥0.45. The Agile 3+ score is a probability-based model that incorporates vibration-controlled transient elastography LSM along with common laboratory parameters, including age, AST, ALT, platelet count, and a history of type 2 diabetes mellitus.^9^^–^^11^ The Agile 3+ score has a similar sensitivity and specificity versus LSM (C-statistics of 0.78) but with lower (8.3 vs. 13.1%) indeterminate results.^10^

### PA assessment

PA was quantified as metabolic equivalent (MET)-minutes per week based on the NHANES Physical Activity Questionnaire, adapted from the Global Physical Activity Questionnaire (GPAQ). Participants reported the number of days per week and average duration (in minutes) per day engaged in vigorous-intensity and moderate-intensity activities in 3 domains: occupational (work-related tasks including paid/unpaid work and household chores), transportation (walking or bicycling), and leisure-time recreational activities. Vigorous-intensity activities were assigned a MET score of 8.0, and moderate-intensity activities, including walking or bicycling for transportation, were assigned a MET score of 4.0. Total MET-minutes per week for each activity were computed by multiplying days per week by duration and corresponding MET scores, and then summing across domains. Participants were then categorized as achieving either high (≥600 MET-min/wk) or low (<600 MET-min/wk) PA. Further details regarding NHANES questionnaire administration and MET assignment can be found on the NHANES website (https://wwwn.cdc.gov/Nchs/Data/Nhanes/Public/2017/DataFiles/PAQ_J.htm).

Participants were categorized into either *high physical activity* (≥600 MET-min/wk) or *low physical activity* (<600 MET-min/wk) groups based on established international guidelines. Specifically, the threshold of 600 MET-min/wk aligns with the World Health Organization’s (WHO) recommendations that adults should engage in at least 150 minutes of moderate-intensity or 75 minutes of vigorous-intensity PA weekly, equivalent to roughly 600 MET-minutes per week (150 min × 4 METs for moderate intensity or 75 minutes × 8 METs for vigorous intensity).^12^^,^^13^ This categorization has been widely adopted in epidemiological research examining associations between PA and health outcomes.^14^^,^^15^ Notably, approximately half of the participants in NHANES legitimately reported zero minutes of both LTPA and WPA, resulting in a zero-inflated distribution that cannot be adequately addressed through traditional data transformations such as log transformation.^16^ Therefore, the simplest and most statistically appropriate approach is to analyze PA as a binary variable.

### Survey design and statistical analysis

NHANES employs a complex, multistage, stratified sampling design to ensure nationally representative estimates. Survey-specific methods were used to account for sampling weights, strata, and primary sampling units. A survey design object was created in R using the svydesign function. Logistic regression models (svyglm) with a quasibinomial family examined associations between work-related and leisure-related MET-minutes per week and advanced fibrosis.

Among the confounders considered, dietary intake is crucial yet challenging to accurately adjust for using standard statistical methods due to compensatory dietary responses. Therefore, we applied unsupervised clustering analysis based on 2-day dietary recall data (including percentage calories from protein, carbohydrates, sugar, fiber, alcohol, saturated fats, monounsaturated fats, and polyunsaturated fats) to categorize NHANES participants into 4 distinct dietary patterns: general diet, high-fat low-carbohydrate diet, low-fat high-carbohydrate diet, and high-alcohol diet. While detailed analyses of dietary patterns in relation to SLD subtypes will be reported separately, the derived dietary clusters were utilized as confounding variables in this analysis. Other confounder variables considered include age (rounded to the nearest decade to avoid collinearity with the outcome variable), sex, race/ethnicity, education level, income-to-poverty ratio, SLD etiology, the number of metabolic risk factors, caffeine intake, and smoking. Metabolic risk factors were defined as the presence of any of the following: overweight or obesity, defined as a body mass index ≥25 kg/m2; impaired glucose metabolism, indicated by self-reported diabetes, a fasting glucose level ≥100 mg/dL, or an HbA1c ≥5.7%; low HDL cholesterol, defined as <40 mg/dL for men or <50 mg/dL for women; elevated triglycerides, defined as ≥150 mg/dL; and hypertension, defined as either a self-reported diagnosis, systolic blood pressure ≥130 mm Hg, diastolic blood pressure ≥85 mm Hg, or having been told by a health professional that one has high blood pressure.

Participant characteristics were stratified based on whether their work-related and recreational exercise exceeded 600 MET-minutes. Continuous variables were compared using svymean() to estimate survey-weighted means and svyvar() to estimate survey-weighted variances. Group comparisons for continuous variables were conducted using svyttest() to account for the complex survey design. Categorical variables were summarized as survey-weighted proportions using svyby() and compared between groups with svychisq() to assess differences across categories. All analyses incorporated sample weights, clustering, and stratification to ensure nationally representative estimates. A 2-tailed *p* value of <0.05 was considered statistically significant. Logistic regression analysis was used for the primary outcome, at-risk advanced fibrosis (defined as an Agile 3+ score ≥0.45), and conducted accounting for the NHANES complex survey design, including stratification, clustering, and sampling weights.

## RESULTS

### Study population

A total of 4050 participants with SLD were included in the analysis (Figure 1). The subtypes of SLD were MASLD (n=2236), MetALD (n=1355), and ALD (n=457). Subjects performing ≥600 versus <600 METs/wk for both LTPA and WPA were younger, males, with college or higher education, and had higher income above the poverty threshold. In contrast, subjects with a higher number of CMRF more often performed <600 METs/wk LTPA and WPA. Regarding the subtype of SLD, subjects with MetALD and ALD more often and those with MASLD less often performed ≥600 METs/wk LTPA and WPA (Table 1). Further, the noninvasive test scores like LSM on transient elastography, Agile 3+ score, and controlled attenuation parameter were lower in subjects with ≥600 versus <600 METs/wk of both LTPA and WPA (Table 1).

**TABLE 1:** Baseline characteristics of study participants stratified by physical activity level (<600 vs. ≥600 METs/wk)

		Leisure-time physical activity			Work physical activity		
Variable	Category	<600 MET/wk	≥600 MET/wk	*p*	<600 MET/wk	≥600 MET/wk	*p*
Mean age (SD), y		52.2 (16.2)	48.2 (16.7)	<0.0001	53.0 (16.3)	48.4 (16.3)	<0.0001
Education (%)	<9th grade	4.8	2.0		4.3	3.3	
	9th–11th grade	8.7	4.5		7.0	7.6	
	High school	32.5	23.2	<0.0001	26.4	33.8	<0.0001
	Some college	31.5	29.6		28.8	33.2	
	College graduate	22.5	40.8		33.5	23.1	
Income		2.9	3.4	<0.001	3.2	2.9	<0.004
Females (%)		51.3	34.1	<0.0001	52.4	38.4	0.00011
Overweight (%)		93.8	94.2	0.77	95.1	92.7	0.50
T2DM (%)		58.9	51.1	<0.011	58.6	53.8	<0.022
Hypertension (%)		55.1	44.3	<0.0004	52.8	50.3	0.27
High TG (%)		14.5	13.8	0.70	13.0	15.6	0.070
Low HDL (%)		37.6	32.2	0.019	35.7	35.9	0.91
CMRF (%)	1	18.5	20.4		16.4	25.5	
	2	30.6	33.2		31.4	32.8	
	3	32.6	27.8	<0.0001	32.4	26.1	<0.0001
	4	14.4	15.4		16.1	12.3	
	5	3.9	3.3		3.8	3.3	
Diet cluster (%)	General	43.6	47.8		46.7	43.0	
	High-carb low-fat	21.2	18.5	0.037	19.7	20.9	0.056
	Low-carb high-fat	26.3	21.2		25.3	23.8	
	High alcohol	8.9	12.6		8.3	12.2	
SLD subtype	MASLD	54.6	40.5		55.9	43.6	
	MetALD	34.4	43.5	<0.0001	35.0	40.0	<0.0001
	ALD	11.0	16.1		9.1	16.5	
Advanced fibrosis (%)		26.1	14.9	<0.0001	24.1	20.2	<0.10
TE (kPa), mean (SD)		6.9 (6.3)	6.1 (5.1)	0.014	6.5 (5.3)	6.8 (6.5)	0.160

Abbreviations: ALD, alcohol-associated liver disease; CAP, controlled attenuation parameter; CMRF, cardiometabolic risk factor; MASLD, metabolic dysfunction–associated SLD; MetALD, metabolic dysfunction and alcohol-associated liver disease; SLD, steatotic liver disease; T2DM, type 2 diabetes mellitus; TE, transient elastography; TG, triglycerides.

### PA

**FIGURE 2:**
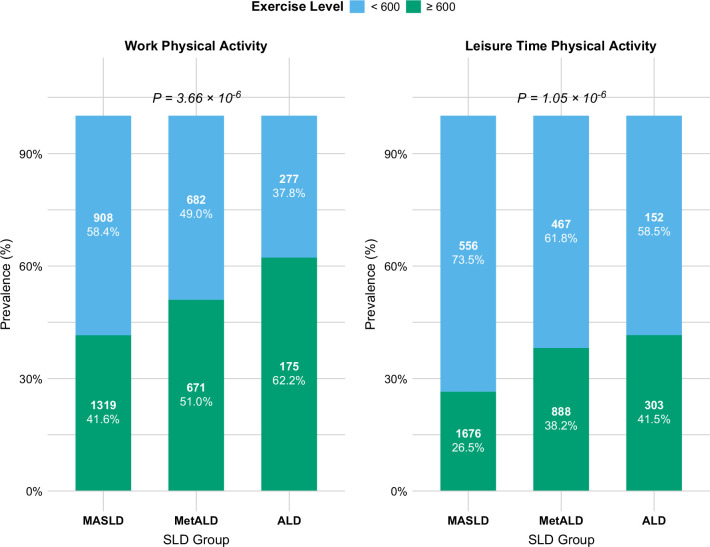
Proportion of individuals engaging in ≥600 METs/wk of physical activity across each SLD group. The number of patients does not match the percentage due to the NHANES survey design. Abbreviations: MET, metabolic equivalent; NHANES, National Health and Nutrition Examination Survey; SLD, steatotic liver disease.

A total of 1867 (47.7%) subjects performed ≥600 METs of WPA. The proportion of subjects performing ≥600 of WPA was 41.6% in MASLD, 51.0% in MetALD, and 62.2% in ALD. Similarly, a total of 1176 (32.8%) subjects performed ≥600 METs of LTPA. The proportion of subjects performing ≥600 minutes of LTPA was 26.5% in MASLD, 38.2% in MetALD, and 41.5% in ALD (Figure 2).

### Advanced fibrosis

**FIGURE 3:**
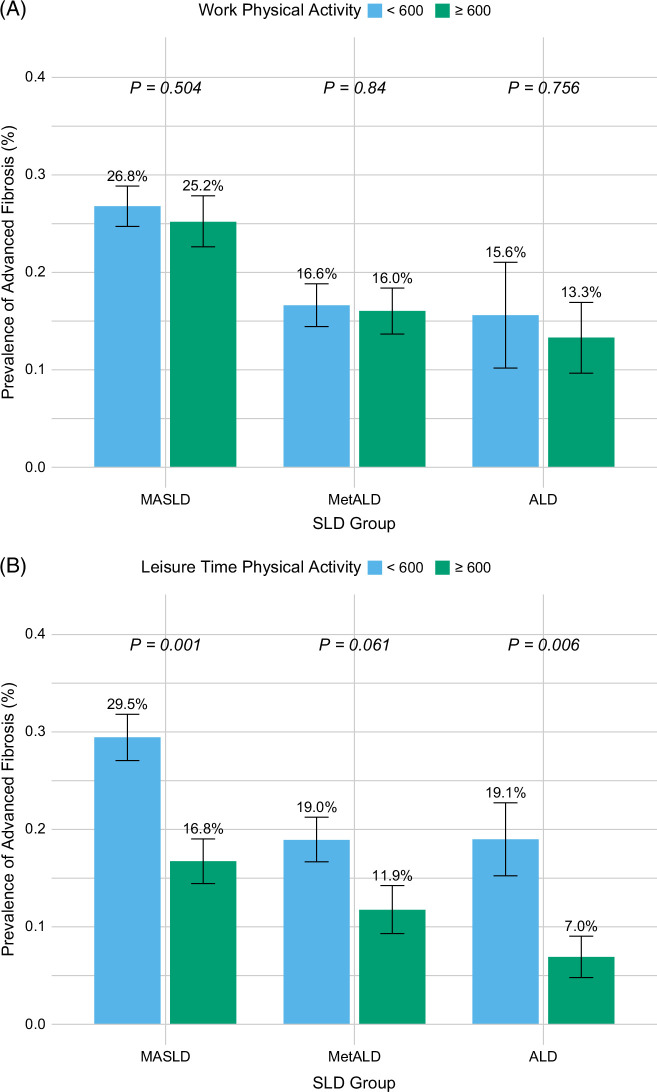
(A) Prevalence of advanced fibrosis by WPA and (B) LTPA across steatotic liver disease groups. Abbreviations: LTPA, leisure-time physical activity; MASLD, metabolic dysfunction–associated steatotic liver disease; MetALD, metabolic dysfunction and alcohol-associated liver disease; SLD, steatotic liver disease; WPA, work physical activity.

The prevalence of at-risk advanced fibrosis was lower among subjects performing ≥600 versus <600 METs/wk WPA (19.6% vs. 22.1%, *p*<0.001), with 14% reduced odds (OR: 0.86, 95% CI: 0.71–1.03) of advanced fibrosis (Figures 3A, 4A). In a multivariable logistic regression model controlled for age, sex, race/ethnicity, education level, income-to-poverty ratio, SLD etiology, and the number of CMRFs, the odds for advanced fibrosis with ≥600 versus <600 METs/wk of WPA was similar (OR: 1.02, 95% CI: 0.77–1.35) (Figure 4B). Similarly, the prevalence of at-risk advanced fibrosis was lower among subjects performing ≥600 versus <600 METs/wk LTPA (13.1% vs. 24.7%, *p*<0.0001) with 54% reduced odds (OR: 0.46, 95% CI: 0.34–0.62) of advanced fibrosis in unadjusted analysis (Figures 3B, 4C) and 43% reduced odds (OR: 0.57, 95% CI: 0.39–0.82) in adjusted analysis (Figure 4D).

FIGURE 4Association between WPA and advanced fibrosis across steatotic liver disease groups for unadjusted (A) and adjusted analyses (B). (C, D) Association between LTPA and advanced fibrosis across steatotic liver disease groups for unadjusted and adjusted analyses, respectively. The adjusted analyses controlled for demographics (age, sex, income-to-poverty ratio, education), number of cardiometabolic risk factors, caffeine intake, diet cluster, and smoking. Abbreviations: ALD, alcohol-associated liver disease; LTPA, leisure-time physical activity; MASLD, metabolic dysfunction–associated steatotic liver disease; MetALD, metabolic dysfunction and alcohol-associated liver disease; SLD, steatotic liver disease; WPA, work physical activity.

**Figure:**
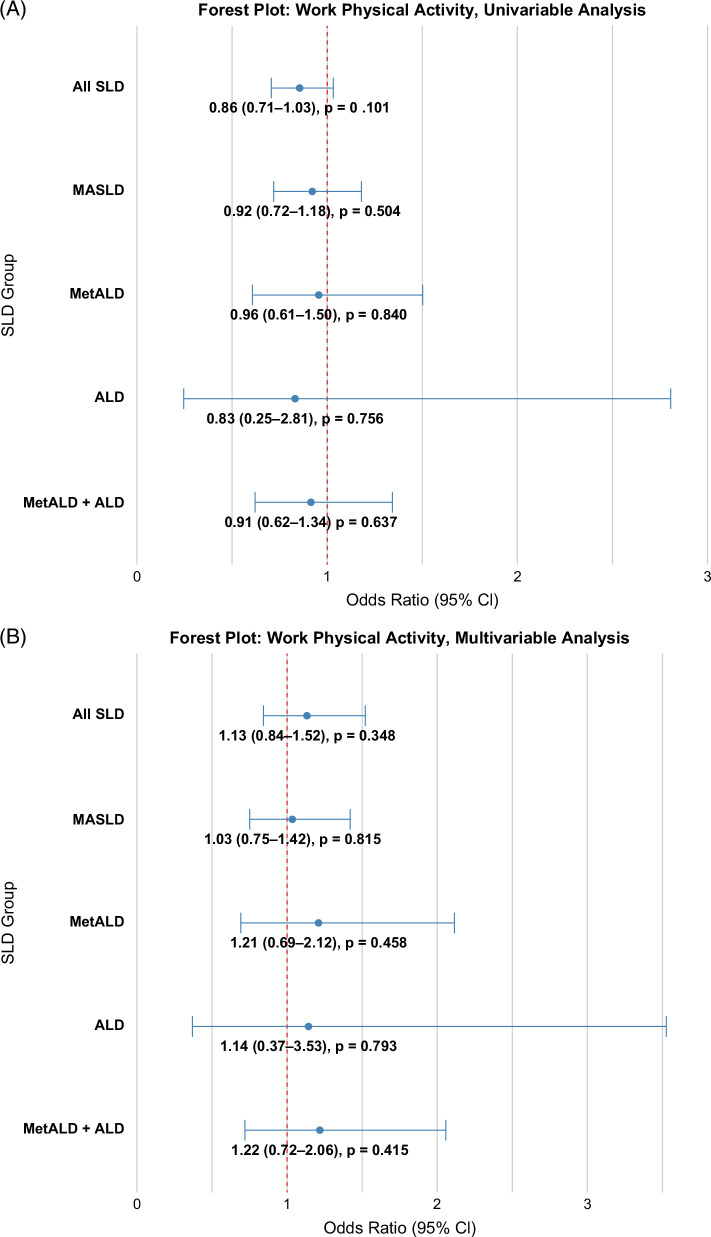

**Figure:**
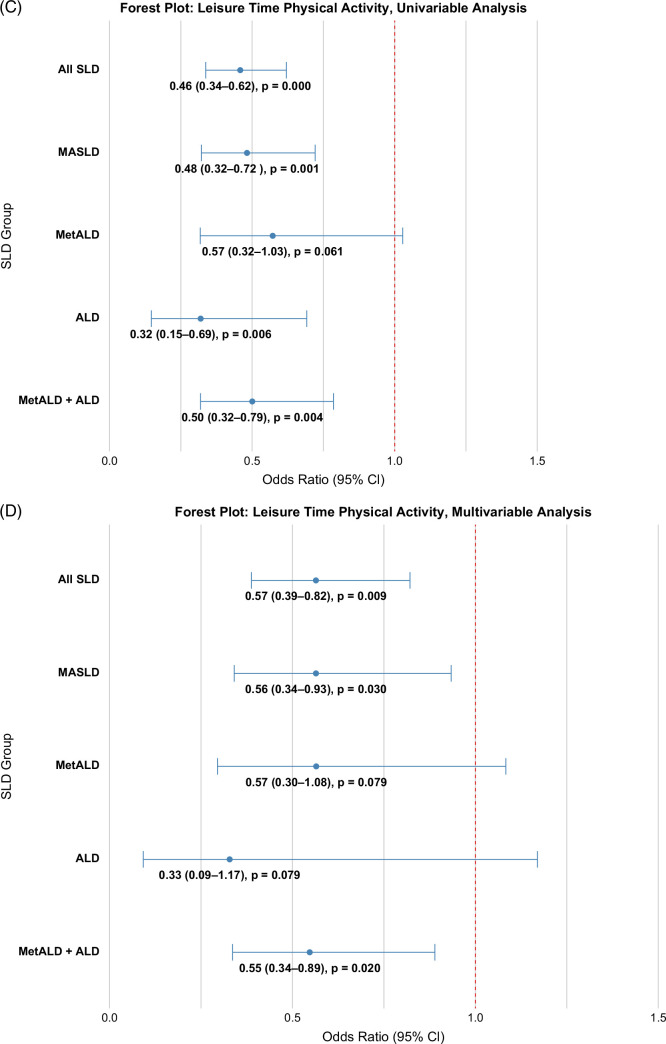

On a univariate analysis, age and number of CMRF were associated with increased odds for advanced fibrosis, with respective OR (95% CI) of 1.07 (1.06–1.08) and 1.88 (1.72–1.94), respectively. In contrast, MetALD and ALD versus MASLD, Hispanic and Asian versus White race, and education level were associated with lower odds for advanced fibrosis (Supplemental Figure S1, Supplemental Digital Content 1, http://links.lww.com/HC9/C109).

### SLD subtype and PA

#### Interactions

The testing for interaction between WPA and SLD subtypes (*p*=0.98) and LTPA and SLD subtypes (*p*=0.55) was negative, indicating there is no evidence of difference among the SLD subtypes. For comprehensiveness, we proceeded to explore the point estimate of each subtype.

#### MASLD

The prevalence of at-risk advanced fibrosis comparing ≥600 versus <600 METs/wk WPA was 25.2% versus 26.8% (Figure 3A), with no difference (OR: 0.86, 95% CI: 0.71–1.03) of advanced fibrosis in unadjusted analysis (Figure 4A) and in adjusted analysis, (OR: 1.03, 95% CI: 0.75–1.72) (Figure 4B). The prevalence of at-risk advanced fibrosis comparing ≥600 versus <600 METs/wk LTPA was 29.5% versus 16.8% (Figure 3B), with (OR: 0.48, 95% CI: 0.32–0.72) in unadjusted analysis (Figure 4C) and (OR: 0.56, 95% CI: 0.34–0.93) in adjusted analysis (Figure 4D).

#### MetALD

The prevalence of at-risk advanced fibrosis comparing ≥600 versus <600 METs/wk WPA was 16.0% versus 16.6% (Figure 3A), with no difference (OR: 0.92, 95% CI: 0.72–1.18) of advanced fibrosis in unadjusted analysis (Figure 4A) and in adjusted analysis, (OR: 1.21, 95% CI: 0.69–2.11) (Figure 4B). The prevalence of at-risk advanced fibrosis comparing ≥600 versus <600 METs/wk LTPA was 11.9% versus 19.0% (Figure 3B), with (OR: 0.57, 95% CI: 0.32–1.03) in unadjusted analysis (Figure 4C) as well as in adjusted analysis (OR: 0.57, 95% CI: 0.68–1.09) (Figure 4D).

#### ALD

The prevalence of at-risk advanced fibrosis comparing ≥600 versus <600 METs/wk WPA was 13.3% versus 15.6% (Figure 3A), with no difference (OR: 0.95, 95% CI: 0.61–1.50) of advanced fibrosis in unadjusted analysis (Figure 4A) and in adjusted analysis, (OR: 1.14, 95% CI: 0.37–3.53) (Figure 4B). The prevalence of at-risk advanced fibrosis comparing ≥600 versus <600 METs/wk LTPA was 7.0% versus 19.1% (Figure 3B), with (OR: 0.32, 95% CI: 0.15–0.69) in unadjusted analysis (Figure 4C) as well as in adjusted analysis (OR: 0.33, 95% CI: 0.09–1.17) (Figure 4D).

#### MetALD and ALD combined

The adjusted OR for WPA ≥600 METs/wk was 1.22 (95% CI: 0.72–2.06). The adjusted OR for LTPA ≥600 METs/wk was 0.55 (95% CI: 0.34–0.89).

In the adjusted multivariable regression models for all subtypes of SLD in both WPA and LTPA analyses, consistent variables associated with increased odds for advanced fibrosis were age and number of CMRFs. In the LTPA analysis, Hispanic versus Caucasian race was associated with lower odds for advanced fibrosis for all individuals with any SLD and for MetALD subtype (Supplemental Table S1, Supplemental Digital Content 2, http://links.lww.com/HC9/C110). Similarly, in the WPA analysis, Hispanic versus Caucasian race and education level were associated with lower odds for advanced fibrosis for individuals with MetALD subtype (Supplemental Table S2, Supplemental Digital Content 2, http://links.lww.com/HC9/C110).

## DISCUSSION

Within this NHANES cohort, we identified substantial cross-sectional associations between PA and advanced fibrosis among individuals with SLD. Further, these associations differed by type of activity, with LTPA but not WPA independently associated with the reduced odds of advanced fibrosis in MASLD and MetALD. Although our sample was too small to demonstrate an independent effect of LTPA in individuals with ALD, numerical trends suggest that LTPA may confer similar protective benefits on advanced fibrosis. The results demonstrate that LTPA is a modifiable risk factor in treating advanced fibrosis in SLD.

The beneficial impact of PA on liver health is well established in mitigating steatosis in those at risk for MASLD and in reducing the risk of advanced fibrosis in those with established MASLD. LTPA has been shown to reduce liver fat content, improve biochemical markers, and risk of advanced fibrosis in patients with MASLD. This beneficial effect is independent of weight loss, with aerobic exercise showing the potential for rapid improvement in hepatic steatosis and biochemical markers even with short-term intervention.^1^ Longer programs of 4 weeks to 6 months duration are associated with even more benefits, like a decrease in intrahepatic triglyceride content, insulin resistance, and histological improvement in MASLD.^17^^–^^24^ In another report, ≥150 min/wk of moderate-intensity LTPA prevented MASLD with additive effects from resistance exercise.^3^ In an Israeli National Health and Nutrition Survey, it was also observed that regular LTPA, especially anaerobic, was associated with less severe steatosis per SteatoTest.^25^

Besides improving liver outcomes, observational studies support the benefit of LTPA in improving patient survival. For example, the long-term follow-up of NHANES cohorts in one study showed that LTPA was associated with a lower risk of mortality among patients with MASLD.^2^^,^^6^ In addition, vigorous LTPA also seems to have other protection against all-cause mortality, as shown in a previous NHANES cohort with longer mortality follow-up.^5^ Despite strong findings in MASLD, the impact of LTPA on MetALD and ALD is unknown. Our study fills the knowledge gap of similar benefits in individuals with MetALD or ALD. In our study, LTPA was associated with a beneficial effect of reducing odds of advanced fibrosis across the whole SLD spectrum, with a very significant risk reduction in MASLD and MetALD and a trend toward benefit in ALD. The absence of a statistically significant correlation in ALD can be due to a smaller number of observations or different injury mechanisms in ALD.

Our study demonstrates that LTPA but not WPA was linked with an independent effect to benefit in reducing the odds of advanced fibrosis in any SLD subgroup. Several large-scale studies across diverse cardiometabolic outcomes have consistently demonstrated that LTPA confers protective health benefits, whereas WPA does not—a phenomenon known as the “physical activity paradox.” For example, the Copenhagen General Population Study revealed that high LTPA significantly reduces cardiovascular and all-cause mortality, whereas elevated WPA is linked to increased risks.^26^ Similarly, UK Biobank and NANES analyses have also shown mortality benefits from LTPA but minimal or no protective effects from occupational activity.^27^^–^^29^ This paradox has also been observed for type 2 diabetes incidence and hypertension, where LTPA consistently correlates with reduced risk, while WPA does not confer similar protection.^30^^,^^31^ Potential mechanisms underlying this discrepancy include that LTPA typically involves structured intensity, adequate recovery periods, and voluntary engagement, which collectively promote beneficial metabolic adaptations, improved mitochondrial function, reduced oxidative stress, and lower systemic inflammation. In contrast, WPA is often characterized by prolonged exertion with inadequate recovery and sustained physiological stress, leading to elevated chronic inflammation, increased oxidative stress, and impaired cardiovascular and metabolic health.^32^^,^^33^ Our results in liver fibrosis are consistent with current literature in cardiometabolic ourcomes.^34^

Our study provides novel data on the benefits of LTPA across the SLD spectrum. Other strengths are the use of a database representative of the US population and controlling for socioeconomic status, which can be associated with the type and intensity of work and related stress.^34^ We used education and household income as surrogate variables of socioeconomic status, and measured the outcome of fibrosis using a more accurate noninvasive test of AGIL3+ score.^34^ However, we recognize certain limitations of our study. First, a cross-sectional observational design limits the determination of causality and generalizability of our findings. Further, the PA was self-reported without detailed information on the quality and intensity of PA, which could have resulted in misclassification of PA levels. The database was also unable to provide information on the total energy expenditure in the 2 different types of PA. In addition, SLD subtypes were categorized by self-reported alcohol use, which can be affected by underreporting of alcohol use due to recall bias, the sick-quitter effect, and stigma.^35^ Despite these limitations, our findings encourage the prescription of PA in MASLD and MetALD, as well as the implementation of sustained, population-wide, best-practice communication campaigns to promote PA among the overall population.^36^

In conclusion, this study clearly demonstrates the differential impact of PA types on advanced liver fibrosis in patients across all subtypes of SLD, including MASLD, MetALD, and ALD. LTPA consistently showed a significant protective effect against advanced fibrosis in patients with MASLD and MetALD, and a similar beneficial trend in ALD. In contrast, WPA did not confer protection against advanced fibrosis after adjusting for relevant confounders, underscoring a critical distinction between structured leisure exercise and occupational PA. These findings highlight the importance of promoting structured leisure-time exercise rather than relying solely on occupational activity to mitigate fibrosis risk in patients with SLD. Further prospective research and interventional studies are necessary to confirm these findings and optimize PA recommendations tailored specifically to the diverse SLD patient population.

## Supplementary Material

## References

[1] Montesi L Moscatiello S Malavolti M Marzocchi R Marchesini G. Physical activity for the prevention and treatment of metabolic disorders. Intern Emerg Med. 2013;8:655–666. 23657989 10.1007/s11739-013-0953-7

[2] Curci R Bonfiglio C Franco I Bagnato CB Verrelli N Bianco A. Leisure-time physical activity in subjects with metabolic-dysfunction-associated steatotic liver disease: An all-cause mortality study. J Clin Med. 2024;13:3772. 38999337 10.3390/jcm13133772 PMC11242783

[3] Park JH Lim NK Park HY. Protective effect of leisure-time physical activity and resistance training on nonalcoholic fatty liver disease: A nationwide cross-sectional study. Int J Environ Res Public Health. 2022;19:2350. 35206539 10.3390/ijerph19042350 PMC8872481

[4] Heinle JW DiJoseph K Sabag A Oh S Kimball SR Keating S. Exercise Is medicine for nonalcoholic fatty liver disease: Exploration of putative mechanisms. Nutrients. 2023;15:2452. 37299416 10.3390/nu15112452 PMC10255270

[5] Henry A Paik JM Austin P Eberly KE Golabi P Younossi I. Vigorous physical activity provides protection against all-cause deaths among adults patients with nonalcoholic fatty liver disease (NAFLD). Aliment Pharmacol Ther. 2023;57:709–722. 36380111 10.1111/apt.17308

[6] Wang X Wang A Zhang R Cheng S Pang Y. Associations between healthy lifestyle and all-cause mortality in individuals with metabolic associated fatty liver disease. Nutrients. 2022;14:4222. 36296904 10.3390/nu14204222 PMC9609442

[7] Alkhouri N Almomani A Le P Payne JY Asaad I Polanco P. The prevalence of metabolic dysfunction-associated steatotic liver disease (MASLD)-related advanced fibrosis and cirrhosis in the United States population utilizing AGILE 3 + and AGILE 4 scores: Analysis of the NHANES 2017-2018 cycle. BMC Gastroenterol. 2024;24:218. 38977950 10.1186/s12876-024-03295-8 PMC11229262

[8] Dunn N Al-khouri N Abdellatif I Singal AK. Metabolic dysfunction and alcohol-associated liver disease: A narrative review. Clin Transl Gastroenterol. 2025;16:e00828. 39969015 10.14309/ctg.0000000000000828 PMC12101925

[9] Sanyal AJ Foucquier J Younossi ZM Harrison SA Newsome PN Chan WK. Enhanced diagnosis of advanced fibrosis and cirrhosis in individuals with NAFLD using FibroScan-based Agile scores. J Hepatol. 2023;78:247–259. 36375686 10.1016/j.jhep.2022.10.034 PMC10170177

[10] Pennisi G Enea M Pandolfo A Celsa C Antonucci M Ciccioli C. AGILE 3+ score for the diagnosis of advanced fibrosis and for predicting liver-related events in NAFLD. Clin Gastroenterol Hepatol. 2023;21:1293–1302. e5. 35842119 10.1016/j.cgh.2022.06.013

[11] Noureddin M Truong E Alnimer L Gornbein JA Yang JD Alkhouri N. Increased accuracy in identifying NAFLD with advanced fibrosis and cirrhosis: Independent validation of the Agile 3+ and 4 scores. Hepatol Commun. 2023;7. 10.1097/HC9.0000000000000055 PMC10162783 37141504

[12] Organization WH. WHO Guidelines on Physical Activity and Sedentary Behaviour. U.S. National Center for Health Statistics (NCHS); 2020.

[13] Bull FC Al-Ansari SS Biddle S Borodulin K Buman MP Cardon G. World Health Organization 2020 guidelines on physical activity and sedentary behaviour. Br J Sports Med. 2020;54:1451–1462. 33239350 10.1136/bjsports-2020-102955 PMC7719906

[14] Pan M Zou Y Wei G Zhang C Zhang K Guo H. Moderate-intensity physical activity reduces the role of serum PFAS on COPD: A cross-sectional analysis with NHANES data. PLoS One. 2024;19:e0308148. 39110698 10.1371/journal.pone.0308148 PMC11305543

[15] Diao X Ling Y Zeng Y Wu Y Guo C Jin Y. Physical activity and cancer risk: A dose-response analysis for the Global Burden of Disease Study 2019. Cancer Commun (Lond). 2023;43:1229–1243. 37743572 10.1002/cac2.12488 PMC10631483

[16] Min YA A A. Modeling nonnegative data with clumping at zero: A survey. J Iran Stat Soc (JIRSS). 2002;1(1-2):7–33.

[17] van der Heijden GJ Wang ZJ Chu ZD Sauer PJJ Haymond MW Rodriguez LM. A 12-week aerobic exercise program reduces hepatic fat accumulation and insulin resistance in obese, Hispanic adolescents. Obesity (Silver Spring). 2010;18:384–390. 19696755 10.1038/oby.2009.274

[18] Promrat K Kleiner DE Niemeier HM Jackvony E Kearns M Wands JR. Randomized controlled trial testing the effects of weight loss on nonalcoholic steatohepatitis. Hepatology. 2010;51:121–129. 19827166 10.1002/hep.23276 PMC2799538

[19] St George A Bauman A Johnston A Farrell G Chey T George J. Independent effects of physical activity in patients with nonalcoholic fatty liver disease. Hepatology. 2009;50:68–76. 19444870 10.1002/hep.22940

[20] Baba CS Alexander G Kalyani B Pandey R Rastogi S Pandey A. Effect of exercise and dietary modification on serum aminotransferase levels in patients with nonalcoholic steatohepatitis. J Gastroenterol Hepatol. 2006;21(1 pt 1):191–198. 16706832 10.1111/j.1440-1746.2005.04233.x

[21] Sullivan S Kirk EP Mittendorfer B Patterson BW Klein S. Randomized trial of exercise effect on intrahepatic triglyceride content and lipid kinetics in nonalcoholic fatty liver disease. Hepatology. 2012;55:1738–1745. 22213436 10.1002/hep.25548 PMC3337888

[22] Mitchell CJ Churchward-Venne TA West DWD Burd NA Breen L Baker SK. Resistance exercise load does not determine training-mediated hypertrophic gains in young men. J Appl Physiol (1985). 2012;113:71–77. 22518835 10.1152/japplphysiol.00307.2012 PMC3404827

[23] Johnson NA Sachinwalla T Walton DW Smith K Armstrong A Thompson MW. Aerobic exercise training reduces hepatic and visceral lipids in obese individuals without weight loss. Hepatology. 2009;50:1105–1112. 19637289 10.1002/hep.23129

[24] Hu Y Li L Xu Y Yu T Tong G Huang H. Short-term intensive therapy in newly diagnosed type 2 diabetes partially restores both insulin sensitivity and beta-cell function in subjects with long-term remission. Diabetes Care. 2011;34:1848–1853. 21680726 10.2337/dc10-2105 PMC3142020

[25] Zelber-Sagi S Nitzan-Kaluski D Goldsmith R Webb M Zvibel I Goldiner I. Role of leisure-time physical activity in nonalcoholic fatty liver disease: A population-based study. Hepatology. 2008;48:1791–1798. 18972405 10.1002/hep.22525

[26] Holtermann A Schnohr P Nordestgaard BG Marott JL. The physical activity paradox in cardiovascular disease and all-cause mortality: The contemporary Copenhagen General Population Study with 104,046 adults. Eur Heart J. 2021;42:1499–1511. 33831954 10.1093/eurheartj/ehab087 PMC8046503

[27] Pearce M Garcia L Abbas A Strain T Schuch FB Golubic R. Association between physical activity and risk of depression: A systematic review and meta-analysis. JAMA Psychiatry. 2022;79:550–559. 35416941 10.1001/jamapsychiatry.2022.0609 PMC9008579

[28] Golabi P Gerber L Paik JM Deshpande R de Avila L Younossi ZM. Contribution of sarcopenia and physical inactivity to mortality in people with non-alcoholic fatty liver disease. JHEP Rep. 2020;2:100171. 32964202 10.1016/j.jhepr.2020.100171 PMC7490851

[29] Kim D Murag S Cholankeril G Cheung A Harrison SA Younossi ZM. Physical activity, measured objectively, is associated with lower mortality in patients with nonalcoholic fatty liver disease. Clin Gastroenterol Hepatol. 2021;19:1240–1247 e5. 32683103 10.1016/j.cgh.2020.07.023

[30] Stage A Wibaek R Rønn PF Bjørnsbo KS Brøns C Allesøe K. The physical activity health paradox in type 2 diabetes. Am J Prev Med. 2025;68:545–554. 39955155 10.1016/j.amepre.2024.11.018

[31] Byambasukh O Snieder H Corpeleijn E. Relation between leisure time, commuting, and occupational physical activity with blood pressure in 125 402 adults: The Lifelines Cohort. J Am Heart Assoc. 2020;9:e014313. 32067583 10.1161/JAHA.119.014313 PMC7070226

[32] Holtermann A Krause N van der Beek AJ Straker L. The physical activity paradox: Six reasons why occupational physical activity (OPA) does not confer the cardiovascular health benefits that leisure time physical activity does. Br J Sports Med. 2018;52:149–150. 28798040 10.1136/bjsports-2017-097965

[33] Harring M Golabi P Paik JM Shah D Racila A Cable R. Sarcopenia among patients with nonalcoholic fatty liver disease (NAFLD) is associated with advanced fibrosis. Clin Gastroenterol Hepatol. 2023;21:2876–2888. e5. 36848980 10.1016/j.cgh.2023.02.013

[34] Edimo Dikobo SJ Lemieux I Poirier P Després JP Alméras N. Leisure-time physical activity is more strongly associated with cardiometabolic risk than occupational physical activity: Results from a workplace lifestyle modification program. Prog Cardiovasc Dis. 2023;78:74–82. 36565734 10.1016/j.pcad.2022.12.005

[35] Arab JP Diaz LA Rehm J Im G Arrese M Kamath PS. Metabolic dysfunction and alcohol-related liver disease (MetALD): Position statement by an expert panel on alcohol-related liver disease. J Hepatol. 2024;82:744–756. 39608457 10.1016/j.jhep.2024.11.028 PMC12242919

[36] Lazarus JV Mark HE Alkhouri N Díaz LA Duseja A Spearman CW. Best buy interventions to address the burden of steatotic liver disease. Lancet Gastroenterol Hepatol. 2024;9:975–977. 39241795 10.1016/S2468-1253(24)00220-6

