# Supplementary figures and images for: Physical activity and advanced fibrosis in MASLD, MetALD, and ALD in a nationally representative cohort: NHANES 2017–2020
Source: Hepatol Commun. 2025 Oct 14;9(11):e0797. doi: 10.1097/HC9.0000000000000797 (PMC12520209; doi:10.1097/HC9.0000000000000797)

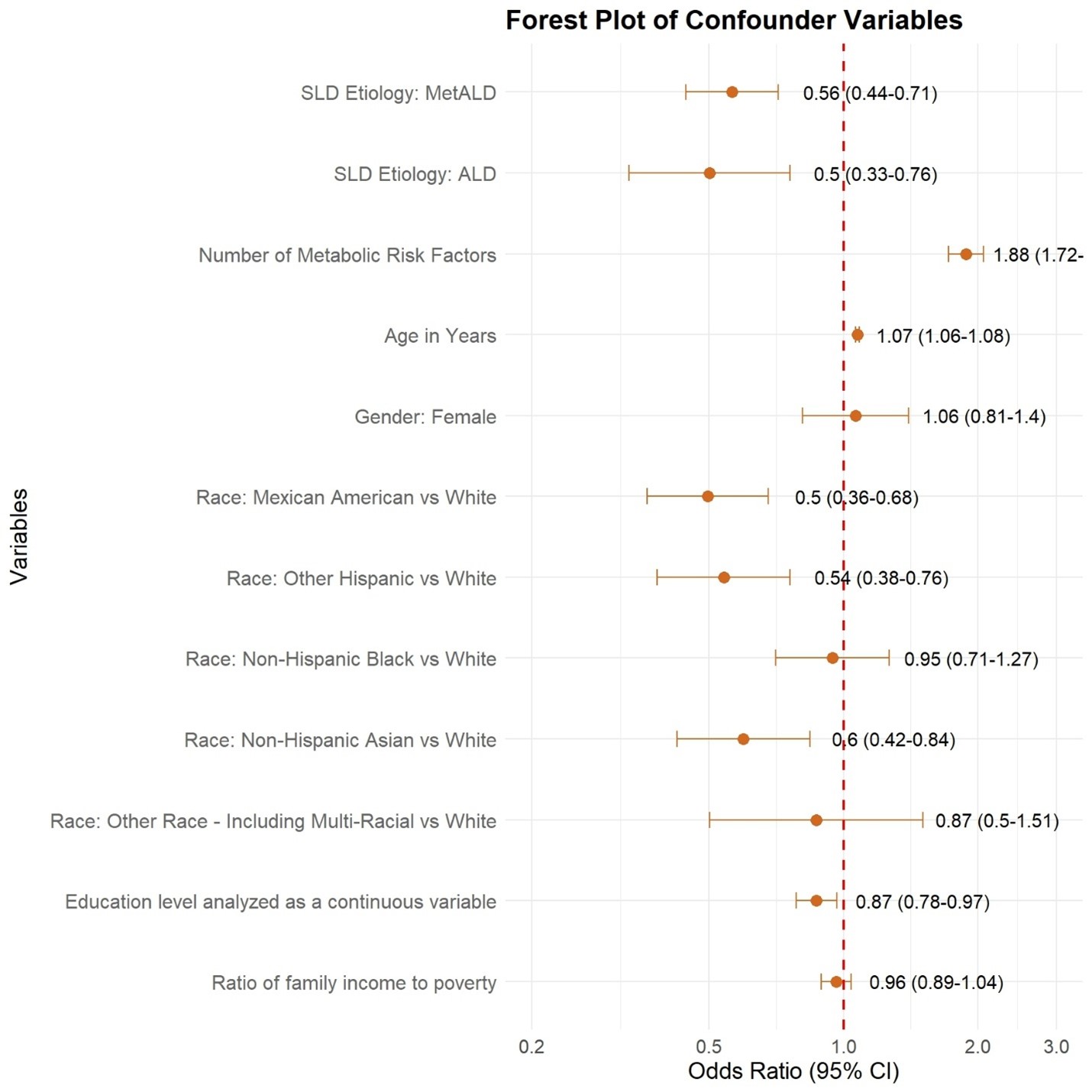

Supplement: Supplementary file 1 [file hc9-9-e0797-s001.jpg]
