# Supplementary material for: Physical activity and advanced fibrosis in MASLD, MetALD, and ALD in a nationally representative cohort: NHANES 2017–2020
Source: Hepatol Commun. 2025 Oct 14;9(11):e0797. doi: 10.1097/HC9.0000000000000797 (PMC12520209; doi:10.1097/HC9.0000000000000797)
Supplement: Supplementary file 2 [file hc9-9-e0797-s002.docx]

**Supplementary Table 1.**

Confounders in Adjusted Multivariable Logistic Regression for Advanced Fibrosis by LTPA Category Across SLD Subtypes.

| Risk Factor | MASLD | MetALD | ALD |
| --- | --- | --- | --- |
| Age (per 10 years) | 1.08 (1.06±1.1) | 1.07 (1.05±1.09) | 1.06 (1.01±1.12) |
| Gender (Male) | 0.68 (0.42±1.11) | 0.74 (0.34±1.63) | 0.91 (0.24±3.5) |
| Race/Ethnicity (Ref = White) |  |  |  |
| Mexican American | 1.34 (0.66±2.73) | 0.56 (0.25±1.27) | 0.61 (0.14±2.7) |
| Other Hispanic | 1.06 (0.66±1.7) | 0.44 (0.21±0.94) | 0.44 (0.11±1.75) |
| Non-Hispanic Black | 1.67 (1.15±2.43) | 0.65 (0.27±1.61) | 0.47 (0.11±1.99) |
| Non-Hispanic Asian | 1.23 (0.79±1.92) | 0.78 (0.39±1.55) | 0.27 (0.02±4.95) |
| Other Race | 1.34 (0.49±3.62) | 0.84 (0.17±4.22) | 0.84 (0.09±7.85) |
| Education level | 0.85 (0.7±1.05) | 1.0 (0.76±1.32) | 2.02 (0.78±5.24) |
| Income/Poverty Ratio | 0.97 (0.79±1.18) | 0.86 (0.73±1.03) | 1.08 (0.79±1.47) |
| Ever Smoked | 1.25 (0.82±1.91) | 0.77 (0.4±1.49) | 1.57 (0.33±7.53) |
| Diet Cluster (Ref = General) |  |  |  |
| High Carb Low Fat | 1.03 (0.59±1.79) | 1.46 (0.61±3.53) | 2.42 (0.45±13.0) |
| High Alcohol | 0.09 (0.01±1.04) | 2.03 (0.82±5.04) | 0.56 (0.09±3.27) |
| Low Carb High Fat | 1.44 (1.05±1.96) | 1.34 (0.71±2.52) | 1.29 (0.25±6.64) |
| Number of CMRF | 1.65 (1.3±2.09) | 1.72 (1.28±2.31) | 2.17 (1.53±3.08) |
| Caffeine intake (g/day) | 1.0 (1.0±1.0) | 1.0 (1.0±1.0) | 1.0 (1.0±1.0) |

**Supplementary Table 2.**
*Confounders in Adjusted Multivariable Logistic Regression for Advanced Fibrosis by WPA Category Across SLD Subtypes.*

| Risk Factor | MASLD | MetALD | ALD |
| --- | --- | --- | --- |
| Age (per 10 years) | 1.08 (1.06±1.1) | 1.07 (1.05±1.1) | 1.07 (1.02±1.12) |
| Gender (Male) | 0.72 (0.45±1.15) | 0.85 (0.38±1.91) | 0.92 (0.26±3.19) |
| Race/Ethnicity (Ref = White) |  |  |  |
| Mexican American | 1.37 (0.66±2.85) | 0.56 (0.24±1.32) | 0.6 (0.14±2.51) |
| Other Hispanic | 1.04 (0.65±1.68) | 0.47 (0.21±1.03) | 0.39 (0.11±1.62) |
| Non-Hispanic Black | 1.65 (1.14±2.39) | 0.68 (0.27±1.67) | 0.44 (0.12±1.62) |
| Non-Hispanic Asian | 1.2 (0.79±1.83) | 0.86 (0.43±1.73) | 0.33 (0.02±4.6) |
| Other Race | 1.42 (0.53±3.79) | 0.77 (0.15±3.89) | 0.94 (0.1±8.51) |
| Education level | 0.84 (0.68±1.03) | 0.98 (0.75±1.27) | 1.8 (0.81±4.01) |
| Income/Poverty Ratio | 0.95 (0.78±1.16) | 0.84 (0.71±0.99) | 1.03 (0.78±1.37) |
| Ever Smoked | 1.25 (0.82±1.92) | 0.79 (0.42±1.49) | 1.4 (0.32±6.13) |
| Diet Cluster (Ref = General) |  |  |  |
| High Carb Low Fat | 1.05 (0.59±1.85) | 1.53 (0.65±3.63) | 2.53 (0.48±13.37) |
| High Alcohol | 0.09 (0.01±1.0) | 1.95 (0.82±4.64) | 0.59 (0.09±3.74) |
| Low Carb High Fat | 1.47 (1.08±2.0) | 1.39 (0.76±2.56) | 1.4 (0.3±6.57) |
| Number of CMRF | 1.68 (1.31±2.15) | 1.72 (1.27±2.33) | 2.13 (1.48±3.05) |
| Caffeine intake (g/day) | 1.0 (1.0±1.0) | 1.0 (1.0±1.0) | 1.0 (1.0±1.0) |
